# Significance of Urinary Full-Length Megalin in Patients with IgA Nephropathy

**DOI:** 10.1371/journal.pone.0114400

**Published:** 2014-12-12

**Authors:** Takuto Seki, Katsuhiko Asanuma, Rin Asao, Kanae Nonaka, Yu Sasaki, Juan Alejandro Oliva Trejo, Hiroyuki Kurosawa, Yoshiaki Hirayama, Satoshi Horikoshi, Yasuhiko Tomino, Akihiko Saito

**Affiliations:** 1 Division of Nephrology, Juntendo University Faculty of Medicine, Tokyo, Japan; 2 TMK project, Medical Innovation Center, Kyoto University Graduate School of Medicine, Kyoto, Japan; 3 Reagents Development Department, Denka Seiken Co. Ltd., Niigata, Japan; 4 Department of Applied Molecular Medicine, Niigata University Graduate School of Medicine and Dental Sciences, Niigata, Japan; University of Bari Aldo Moro, Italy

## Abstract

**Background and Objectives:**

Megalin is highly expressed at the apical membranes of proximal tubular epithelial cells. A urinary full-length megalin (C-megalin) assay is linked to the severity of diabetic nephropathy in type 2 diabetes. This study examined the relationship between levels of urinary C-megalin and histological findings in adult patients with IgA nephropathy (IgAN).

**Design, Setting, Participants, & Measurements:**

Urine samples voided in the morning on the day of renal biopsy were obtained from 73 patients with IgAN (29 men and 44 women; mean age, 33 years) and 5 patients with membranous nephropathy (MN). Renal pathologic variables were analyzed using the Oxford classification of IgAN, the Shigematsu classification and the Clinical Guidelines of IgAN in Japan. The levels of urinary C-megalin were measured by sandwich ELISA.

**Results:**

Histological analysis based on the Oxford classification revealed that the levels of urinary C-megalin were correlated with mesangial hypercellularity in IgAN patients (OR = 1.76, 95% CI: 1.04–3.27, P<0.05). There was a significant correlation between the levels of urinary C-megalin and the severity of chronic extracapillary abnormalities according to the Shigematsu classification in IgAN patients (β = 0.33, P = 0.008). The levels of urinary C-megalin were significantly higher in all risk levels of IgAN patients requiring dialysis using the Clinical Guidelines of IgAN in Japan than in the control group. The levels of urinary C-megalin were significantly higher in the high risk and very high risk grades than in the low risk grade (P<0.05). The levels of urinary C-megalin were significantly higher in MN patients compared to the control group.

**Conclusions:**

The levels of urinary C-megalin are associated with histological abnormalities in adult IgAN patients. There is a possibility that urinary C-megalin is an independent predictor of disease progression of IgAN. In addition, our results suggest that urinary C-megalin is a marker of glomerular abnormalities in various glomerular diseases as well as IgAN.

## Introduction

Megalin is a large (∼600 kDa) glycoprotein member of the low-density lipoprotein receptor family [1], [2] that is highly expressed at the apical membranes of proximal tubular epithelial cells (PTECs) [3]. Megalin plays a central role in the endocytic functions of PTECs [3]. Low-molecular-weight protein markers of PTEC injury, such as α_1_-microglobulin (α_1_-MG) and β_2_-microglobulin (β_2_-MG), are filtered by glomeruli and reabsorbed by PTECs via megalin [4], [5]. Megalin is detected in human urine and increased urinary megalin excretion has been shown in microalbuminuric patients with type 1 diabetes [6]–[9]. Recently, the ectodomain form and the full length form of urinary megalin were evaluated using sandwich ELISA and specific monoclonal antibodies (mAb). An amino-terminal (A-megalin) mAb was used for the ectodomain form, and a carboxyl-terminal (C-megalin) mAb was used for the full length form of megalin [10]. Urinary full-length megalin (C-megalin) levels were found to be linked to the severity of diabetic nephropathy in type 2 diabetes [10].

IgA nephropathy (IgAN) is the most common pattern of primary chronic glomerulonephritis in the world and a diagnosis of IgAN always requires renal biopsy [11]. The histological characteristics of IgAN are mesangial cell proliferation, mesangial matrix expansion, and mesangial IgA deposition [12], [13]. Activated mesangial cells secrete various proinflammatory and profibrotic mediators of renal injury. These mediators cause podocyte injury and PTEC activation, which drives tubulointerstitial abnormalities [14], [15]. Continued immune complex deposition and mesangial cell activation lead to progressive glomerulosclerosis through irreversible podocyte loss [16]. Although IgAN has previously been considered as a benign condition, recent studies indicate that IgAN has the potential for slowly progressive chronic renal impairment, leading eventually to end stage renal disease (ESRD).

Clinical studies of urinary C-megalin have been reported only in patients with diabetes nephropathy. It is still unknown whether urinary C-megalin is associated with renal histological findings in patients with glomerulonephritis such as IgAN. In this study, we focused on urinary C-megalin in adult IgAN patients to understand the relationship between levels of urinary C-megalin and renal histological findings. In addition, we examined the levels of urinary C-megalin in patients with membranous nephropathy (MN).

## Materials and Methods

### Normal sample collection

Urine samples and clinical data were collected from adult residents who had participated in public medical examinations in Tagami-machi (Niigata-ken, Japan) from 2007 to 2009 and from volunteers at Denka Seiken Co., Ltd. (Tokyo, Japan) in 2007 with written informed consent. Normal control individuals (n = 77; 19–65 years of age; male/female  = 40/37) who satisfied standard medical criteria as defined in S1 Table were selected from these populations. Collection of the urine samples and evaluation of clinical data were approved by the ethical committees of Niigata University in accordance with the principles embodied in the Declaration of Helsinki.

### Patients and histological evaluation

From October 2007 to October 2012, urine samples voided on the morning of the day of renal biopsy were obtained from 73 patients with IgAN and 5 patients with membranous nephropathy. The clinical profile of patients with IgAN is shown in Table 1. Renal biopsies were performed on 71 patients with IgAN in Juntendo University Hospital, Tokyo, Japan. The pathologic characteristics of the other two IgAN biopsy specimens were also investigated at Juntendo University Hospital. Patients who were administrated angiotensin converting enzyme inhibitors (ACEI), angiotensin receptor blockers (ARB), and corticosteroid treatment and those who underwent tonsillectomy were excluded from this study. The patients with MN also underwent renal biopsy in Juntendo University Hospital.

**Table 1:** Characteristics of the study subjects.

	control	IgA nephropathy	*p* value
Number (n)	77	73	0.74
Age (years)	30(19–65)	33(18–57)	0.19
Male: Female	40∶37	29∶44	0.13
Serum creatinine (mg/dL)	0.75(0.44–1.04)	0.72(0.44–2.10)	0.88
Urinary protein (g/g.Cr)	0.05(0.03–0.46)	0.37(0.06–2.80)	<0.01
eGFR (ml/min/1.73 m^2^)	87.6(62.3–145.0)	82.8(26.4–155.7)	0.09
Mean blood pressure (mmHg)	77.7(60.3–96.0)	80.0(62.7–112.0)	0.22

Unless otherwise noted, the data are given as median (interquartile range).

eGFR, estimated GFR.

The Shigematsu classification and the Oxford classification were used to evaluate the histological findings of each IgAN case [17]–[19]. To evaluate the histological findings in renal biopsy specimens of the patients, the sections were stained using four stains: hematoxylin-eosin, periodic acid-Schiff, Elastica Masson and periodic acid methenamine silver-Masson trichrome. The histological evaluation of the glomeruli for activity and chronicity was performed according to the method proposed by Shigematsu [17], [20]: noting the extent of extracapillary abnormalities (acute and chronic), endocapillary abnormalities (acute and chronic), and tubulointerstitial abnormalities (acute and chronic). The extent of the chronic glomerular abnormalities was categorized into one of four stages (0, 1, 2, and 3). Regarding the glomerular abnormalities, this evaluation was applied to all the glomeruli in the biopsy specimens, and the average of the scores was taken. These semiquantitative evaluations were processed using statistical analyses. The minimal number of glomeruli evaluated per section was 10 according to the Shigematsu classification.

Furthermore, patients were divided into four dialysis requiring risk levels according to the clinical guides for IgA nephropathy in Japan, third version [21]: (I) low risk, (II) medium risk, (III) high risk, and (IV) very high risk. A dialysis requiring risk level is a combination of a clinical and histological grade. The clinical grade was determined according to the level of urinary total protein excretion and the estimated glomerular filtration rate (eGFR). The histological grade was determined according to percentage of glomeruli with lesions. Glomerular abnormalities were classified as global sclerosis, segmental sclerosis, and crescent formation.

The histological findings of each slide were evaluated by two nephrologists who did not know the details of the patients' clinical data, including the levels of urinary C-megalin.

The analysis of IgAN and MN patients was conducted according to the Declaration of Helsinki and was approved by the Institutional Review Board of Juntendo University Hospital. Informed consent was obtained from all patients in written form. In the cases of children under 19 years old, written consent was obtained from parents, next of kin or from legal guardians. All patients were informed by the clinicians and consent was registered by a signed consent form approved by the Institutional Review Board of Juntendo University Hospital.

### Quantification of urinary C-megalin

Quantification of urinary C-megalin was performed as previously described [10]. In brief, 90 µL of urine was mixed with 10 µL of solution (2 mol/L Tris-HCl, 0.2 mol/L EDTA,10% Triton X-100, pH8.0), and incubated at room temperature for 1 min for the C-megalin assay. After the samples were applied to ELISA plates and immobilized with a capture mAb, the alkaline phosphatase labeled tracer mAb was added to the plate and measured using a chemiluminescent immunoassay detection system. The intra- and interassay coefficient of variation was less than 10%.

### Measurement of other markers

Serum samples from the patients were analyzed in the clinical laboratory center at Juntendo University Hospital. Levels of serum creatinine were measured using standard enzymatic methods. Levels of urinary total protein excretion were measured by the pyrogallol red method using a reagent kit (Protein Assay Rapid Kit, Wako Pure Chemical Industries, Ltd.). Urinary concentrations of creatinine, α_1_-MG, β_2_-MG, and N-acetyl-b-D-glucosaminidase (NAG) were measured by an automated instrument (7170S; Hitachi High-Technologies Corp., Tokyo, Japan) with reagent kits CRE-S (Denka Seiken Co., Ltd.), ALB- TIA 31 (Denka Seiken Co., Ltd.), αMi-Latex (Denka Seiken Co., Ltd.), βMG-Latex (Denka Seiken Co., Ltd.), and N-assay L NAG NITTOBO (Nittobo Medical Co., Ltd., Tokyo, Japan), respectively. The following equation was used to calculate eGFR as described by Matsuo et al. [22]:
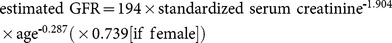



### Immunofluorescence of renal biopsy sections

The renal sections from patients were snap frozen, and the cryosections (3 µm) were post-fixed with cold acetone (−20°C) for 5 min. The renal sections were then blocked using blocking solution [phosphate-buffered saline (PBS), 2% fetal calf serum and 0.2% fish gelatin]. The primary antibodies were incubated at room temperature for 60 min, followed by 30 min of incubation with secondary antibodies at a 1∶300 dilution [Alexa Fluor 488 donkey antirabbit IgG (Invitrogen, Carlsbad, CA)]. Rhodamine phalloidin (Invitrogen, Carlsbad, CA) was incubated for 30 min. Nuclei were stained with DAPI (4′, 6-diamidine-2-phenylin-dole) for 10 min. The slides were washed with PBS several times and then mounted as described before [23]. The samples were analyzed under confocal laser-scanning microscope (Leica SP8 confocal microcopy system, Leica Microsystems, Wetzlar, Germany). Rabbit polyclonal antibodies against human megalin have been described previously [24].

### Statistical analysis

Differences between categorical variables were tested by the chi-square test, quantitative non-parametric variables were tested by the Mann-Whitney U test and quantitative parametric variables were tested by the Welch t-test. Multiple regression analysis with a Stepwise approach was used to test the independent relationship of the classification of the Oxford classification, the Japanese Shigematsu classification, and the levels of urinary total protein excretion, respectively. The significance levels for entering and removing an explanatory variable were set at 0.05 and 0.10 A value of p<0.05 was considered significant. All statistical analyses were performed using JMP version 8.0.1 software (SAS Institute Inc., Cary, NC, USA).

## Results

### Characteristics of the study subjects

Table 1 shows the characteristics of the control and IgAN patients. The levels of urinary total protein excretion in IgAN patients were significantly higher than those in the control group (P<0.01). There was no significant difference in eGFR and mean blood pressure.

### Correlation between the levels of urinary C-megalin, α_1_-MG, β_2_-MG, and NAG and urinary total protein excretion

To assess the correlation between the level of urinary total protein excretion and age, gender, serum creatinine, mean blood pressure, urinary C-megalin, α_1_-MG, β_2_-MG, and NAG, these factors were adopted as explanatory variables in a multiple regression analysis (Table 2). The levels of urinary C-megalin, α_1_-MG, and β_2_-MG were correlated with the levels of urinary total protein excretion. The relationship between the levels of urinary total excretion and each urinary biomarker was as follows: C-megalin, β = 0.18 (p<0.05); β_2_-MG, β = 0.45 (p<0.001); and α_1_-MG, β = 0.40 (P<0.001). The level of NAG was not correlated with the levels of urinary total protein excretion. The levels of urinary C-megalin, α_1_-MG, and β_2_-MG were not correlated with eGFR (S2 Table). The level of NAG was correlated with eGFR (β = −0.27 p<0.01).

**Table 2:** Stepwise multiple regression analysis of levels of urinary total protein excretion with relevant factors.

	IgAN patients(N = 73)	
variables	*β*	*p* value
C-megalin (pmole/g.Cr)	0.18	0.016
β_2_-MG (µg/g.Cr)	0.45	<0.001
α_1_-MG (mg/g.Cr)	0.40	<0.001
NAG(IU/g.Cr)	-	-

C-megalin, full-length megalin; β_2_-MG, β_2_-microglobulin; α_1_-MG, α_1_-microglobulin; NAG, N-acetyl-b-D-glucosaminidase.

### Histological evaluation of renal biopsy specimens

To assess the association between histological abnormalities and age, gender, serum creatinine, mean arterial pressure, urinary C-megalin, α_1_-MG, β_2_-MG, NAG, and urinary total protein, these factors were adopted as explanatory variables in a multiple regression analysis (Tables 3 and 4).

**Table 3:** Stepwise multiple regression analysis of histological variables in the Oxford classification with relevant factors.

	Mesangial hypercellularity		Segmental glomerulosclerosis	
	IgAN patients(N = 59)		IgAN patients(N = 59)	
	OR	*p* value	OR	*p* value
	(95%CI)		(95%CI)	
C-megalin	1.76	0.046	-	-
(pmole/g.Cr)	(1.04–3.27)			
β_2_-MG	-	-	0.98	0.007
(µg/g.Cr)			(0.96–0.99)	
α_1_-MG	-	-	-	-
(µg/g.Cr)				
NAG	-	-	-	-
(IU/g.Cr)				
Urinary total protein	-	-	280.51	0.002
(g/g.Cr)			(13.70–16083.05)	

	Endocapillary hypercellularity		Tubular atrophy/interstitial fibrosis	
	IgAN patients(N = 59)		IgAN patients(N = 59)	
	OR	*p* value	OR	*p* value
	(95%CI)		(95%CI)	
C-megalin	-	-	-	-
(pmole/g.Cr)				
β_2_-MG	-	-	-	-
(µg/g.Cr)				
α_1_-MG	-	-	-	-
(µg/g.Cr)				
NAG	-	-	-	-
(IU/g.Cr)				
Urinary total protein	-	-	-	-
(g/g.Cr)				

IgAN: IgA nephropathy.

**Table 4:** Stepwise multiple regression analysis of histological variables in the Shigematsu classification with relevant factors.

variables	chronic extracapillary abnormalities		chronic endocapillary abnormalities	
	IgAN patients (N = 59)		IgAN patients (N = 59)	
	*β*	*p* value	*β*	*p* value
C-megalin(pmole/g.Cr)	0.33	0.008	0.21	0.085
β_2_-MG (µg/g.Cr)	0.21	0.075	-	-
α_1_-MG (mg/g.Cr)	-	-	-	-
NAG(IU/g.Cr)	-	-	-	-
Urinary total protein(g/g.Cr)	-	-	0.30	0.016

### Correlation of urinary markers with histological variables in the Oxford classification

The levels of urinary C-megalin were correlated with mesangial hypercellularity (OR = 1.76, 95% CI: 1.04–3.27, P = 0.046) (Table 3). The levels of urinary β_2_-MG were correlated with segmental glomerulosclerosis (OR = 0.98, 95% CI: 0.96–0.99, P<0.01). The levels of urinary total protein excretion were correlated with segmental glomerulosclerosis (OR = 280.51 95% CI: 13.70–16083.05, P<0.01). The levels of urinary C-megalin, α_1_-MG, β_2_-MG, and NAG were not correlated with tubular atrophy and interstitial fibrosis. The levels of urinary C-megalin were correlated with mesangial hypercellularity, showing a distinctive clinical significance compared to preexisting urinary biomarkers.

### Correlation of urinary markers with histological variables in the Shigematsu classification

Because the levels of urinary C-megalin showed relevance in the detection of glomerular abnormalities according to the Oxford classification, we enrolled 59 patients in order to determine whether there were also glomerular abnormalities according to the Shigematsu classification [17] (Table 4). There was a significant correlation between the levels of urinary C-megalin and the severity of chronic extracapillary abnormalities (β = 0.33, P = 0.008). The levels of urinary C-megalin had some relevance to chronic endocapillary abnormalities (β = 0.21, P = 0.085). The levels of urinary C-megalin were not correlated with other histological variables used in the Shigematsu classification (data not shown). The levels of urinary β_2_-MG had some relevance to chronic extracapillary abnormalities (β = 0.21, P = 0.075). There was a significant correlation between levels of urinary total protein excretion and the severity of chronic endocapillary abnormalities (β = 0.30, P = 0.016).

### Relationship between levels of urinary C-megalin and dialysis requiring risk levels

**Figure 1:**
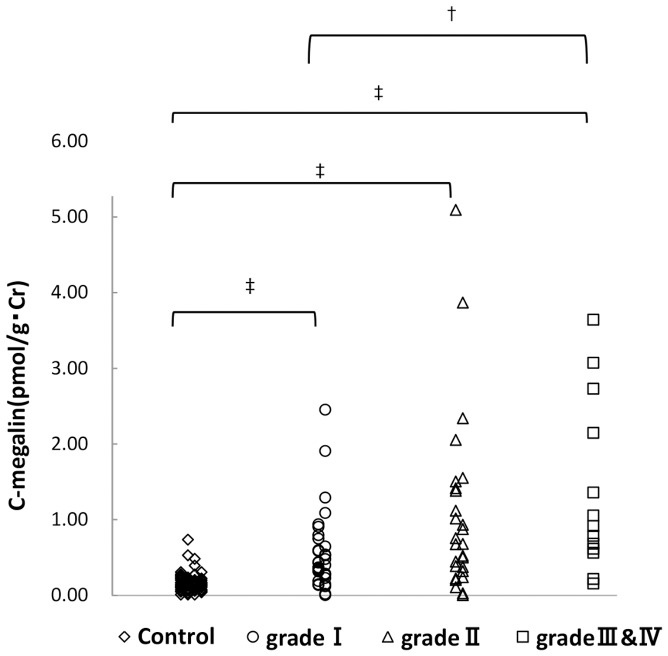
Relationship of risk levels of requiring dialysis to levels of urinary C-megalin. The numbers on horizontal bars means; control, control subjects (N = 77); I, low risk group (N = 33); II, medium risk group (N = 27); III and IV, high and super high risk group (N = 13). †, p<0.05; ‡, p<0.001.

We enrolled 73 patients to in order to analyze the relationships between the level of urinary C-megalin and the dialysis requiring risk levels from the Clinical Guideline for IgAN Patients in Japan, third version [20]. The levels of urinary C-megalin were significantly higher in all grades of IgAN patients than in the control group (Fig. 1). The levels of urinary C-megalin were significantly higher in grades III and IV than in grade I (P<0.05).

### Renal expression of megalin in IgA nephropathy

**Figure 2:**
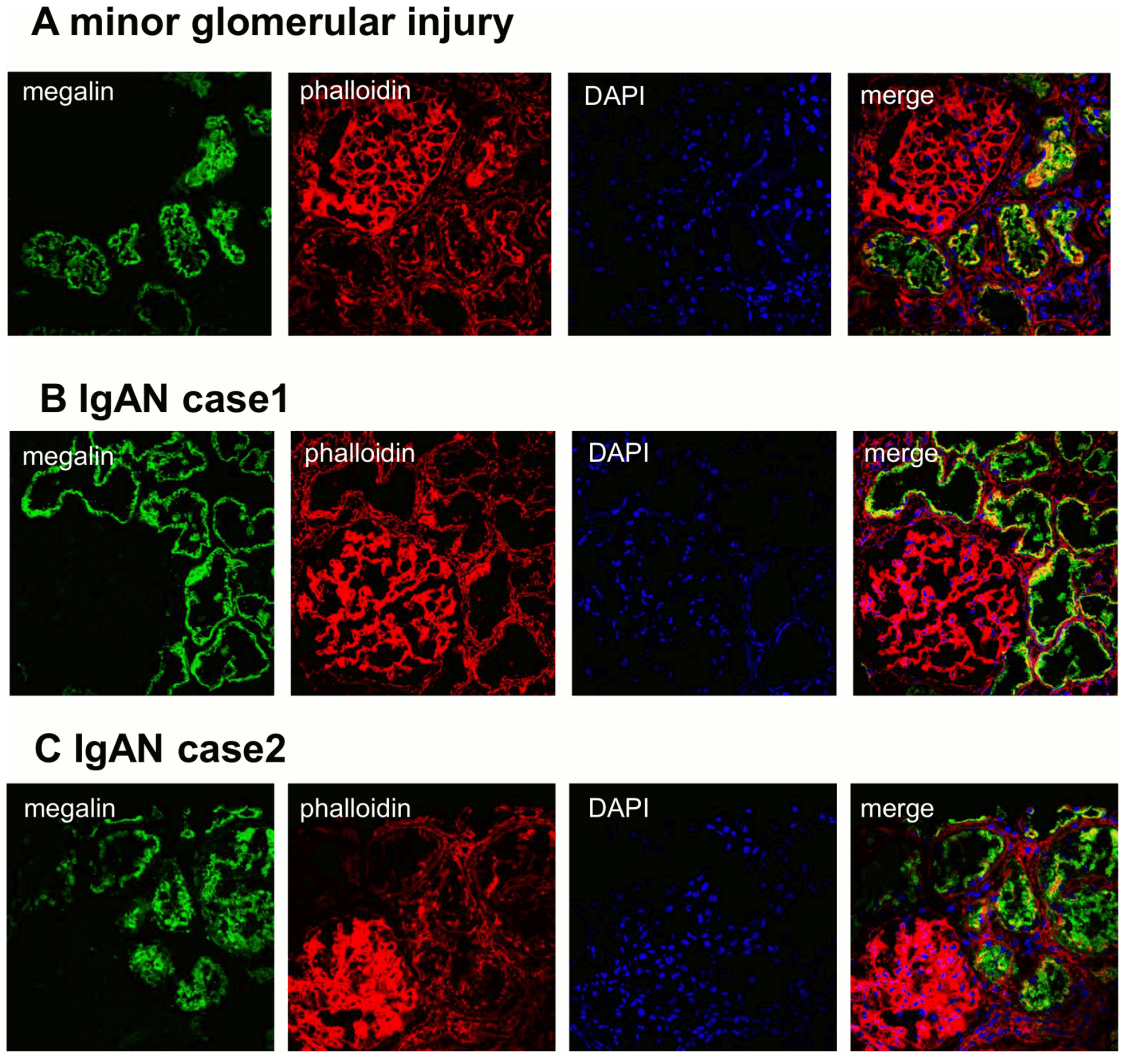
Triple staining for megalin, phalloidin and DAPI in renal biopsy specimens from IgAN patients. Triple staining for megalin, phalloidin and DAPI in renal biopsy specimens from minor glomerular injury patients (A) and IgAN patients (B, C) showed that megalin was localized in the brush border of proximal tubules.

Triple immunostaining of renal biopsy samples for megalin, phalloidin and DAPI are shown in Fig. 2. Megalin was localized in the brush border of proximal tubules but not in the glomerulus in both minor glomeruli injury and IgAN. There was no appreciable difference in the staining pattern of megalin between minor glomerular injury and that of IgAN.

### The levels of urinary C-megalin in patients with MN

**Figure 3:**
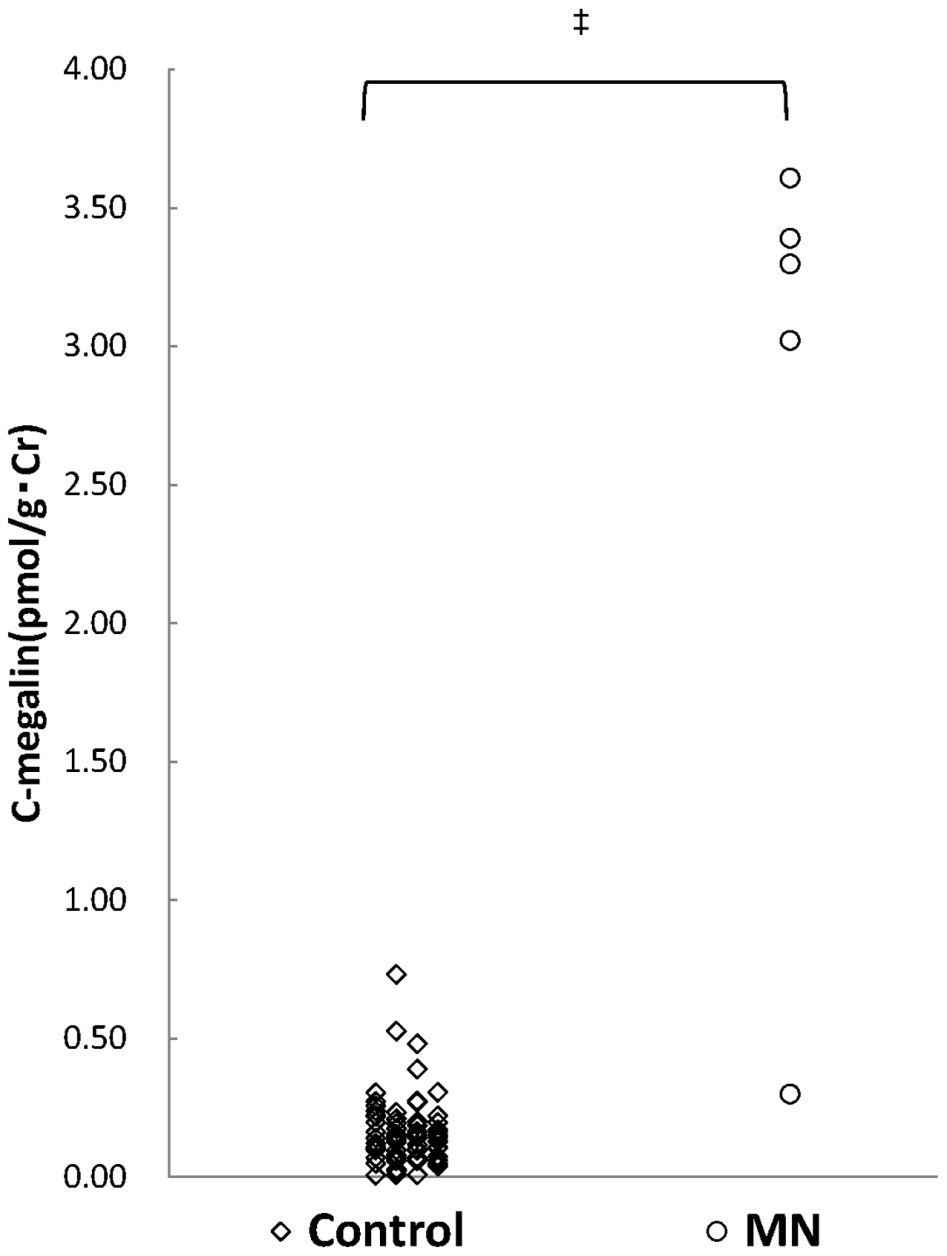
The levels of urinary C-megalin in patients with membranous nephropathy. MN: membranous nephropathy. ‡, p<0.001

To check whether increasing levels of urinary C-megalin are found in other glomerulonephritis, we measured the levels in urine samples from 5 patients with MN. The results showed significantly higher levels of urinary C-megalin in 4 out of 5 MN patients when compared to the control group (Fig. 3), indicating that urinary C-megalin is not specifically increased in patients with IgAN or diabetic nephropathy.

## Discussion

We identified that the levels of urinary C-megalin were correlated with chronic extracapillary abnormalities in IgAN patient groups. In addition, the levels of urinary C-megalin were correlated with mesangial hypercellularity. In humans, megalin was only detected in PTECs [25]. In our study, megalin was also localized at the brush border of proximal tubules but not in glomeruli in both minor glomerular injury and IgAN. There was a possibility that the levels of urinary C-megalin excreted from PTECs were associated with glomerular abnormalities. In the present study, the levels of urinary C-megalin were also significantly higher in the MN patients. The target of injury in MN is the glomerular visceral epithelial cells, or podocytes, beneath which the deposits are formed [26]. What is the mechanism through which levels of urinary C-megalin reflect glomerular abnormalities? There are several proposed mechanisms for urinary C-megalin levels reflecting glomerular abnormalities. The first reason is that glomerular abnormalities trigger PTEC dysfunction. Although it remains unclear how glomerular abnormalities lead to tubulointerstitial injury in IgAN, one of the possible pathogenetic mechanisms is glomerulopodocytic-tubular communication [27]. For example, humoral factors, such as the tumor necrosis factor α and the transforming growth factor β, are released from the activated mesangial cells in order to alter podocyte gene expression and glomerular permeability [14], [27]–[29]. It has been postulated that these mediators from mesangial cells first activate the podocytes before reaching the tubulointerstitium, either by glomerular filtration or by transportation via the postglomerular capillaries [27]. Then, podocyte specific injuries lead to an adhesion to Bowman's capsules and glomerular sclerosis in experimental diseases [30]. These mediators stimulate tubular epithelial cells to produce cytokines and chemokines that eventually induce tubular damage, interstitial mononuclear cells infiltration and fibrosis [15]. The second reason is that there is a possibility that PTEC dysfunction aggravates chronic glomerular abnormalities. Angiotensin II, which is known to induce PTEC hypertrophy, was found to be taken up by PTECs via megalin-mediated endocytosis [31]. The finding suggests that megalin may regulate the action of angiotensin II on PTECs by processing its degradation or transcytosis [32]. Inflammatory cytokines, including angiotensin II, activate PTECs. This amplifies the inflammatory cascade by the local production of chemotactic mediators, which attract even more inflammatory component cells [33]. Grgic *et al*. reported that selective PTEC injury could drive the formation of interstitial fibrosis and potentially glomerulosclerosis [34]. Recently, it has been reported that the renal tubular sirtuin 1 attenuated diabetic albuminuria by epigenetically suppressing claudin-1 overexpression in podocytes [35]. They suggested that sirtuin 1 in PTECs can affect glomerular function. Possibly, the progression of PTEC injury causes aggravation of chronic extracapillary abnormalities and leads to glomerulosclerosis.

Tubulointerstitial abnormalities play a pivotal role in the progression of IgAN [19]. Urinary C-megalin is considered a new biomarker of PTEC injury because megalin is expressed at the apical membranes of PTECs and excreted more in the urine in patients with diabetic nephropathy. However, urinary C-megalin was not correlated with tubular atrophy and interstitial fibrosis in our study. Also, the other urinary biomarkers were not correlated with these tubulointerstitial findings. Peters et al. reported that the tubulointerstitial score correlated with α_1_-MG and β_2_-MG in IgAN [36]. The rate of patients who had severe tubular atrophy and interstitial fibrosis in our study was smaller than in previous study. This could be the reason why urinary C-megalin and other markers were not correlated with tubular atrophy and interstitial fibrosis in our study.

In this study, levels of urinary C-megalin were associated with the dialysis requiring risk levels from the Clinical Guidelines for IgAN in Japan, third version. The Oxford classification of IgAN identified that four pathologic abnormalities independently determine the risk of developing progressive renal disease: mesangial hypercellularity, endocapillary hypercellularity, segmental glomerulosclerosis, and tubular atrophy/interstitial fibrosis [16], [19]. Levels of urinary C-megalin were correlated with mesangial hypercellularity by the Oxford classification. Although urinary levels of β_2_-MG were correlated with segmental glomerulosclerosis, its urinary excretion did not offer an advantage over proteinuria in predicting prognosis in patients with IgAN [36]. Thus, there is a possibility that urinary C-megalin is an independent predictor of disease progression of IgAN. In a future study, in order to determine whether urinary C-megalin is a predictor, we need to perform a longitudinal study to follow up with the patients. Significantly higher levels of urinary C-megalin in patients with diabetic nephropathy[10], IgAN and MN, suggests that urinary C-megalin relates to glomerular abnormalities and disease progression in glomerular diseases. In this study, the number of MN patients was small and the histological findings were not analyzed. To investigate whether urinary C-megalin is related to glomerular abnormalities and disease progression in MN, we need to assess the correlation between the levels of urinary C-megalin and the histological findings, and follow up with the patients.

The levels of urinary C-megalin were correlated with chronic extracapillary abnormalities. According to a review of IgAN, supportive therapy with the cautious use of ACEI or ARB should be continued in order to slow the process, although an eGFR that is persistently less than 30 mL/min/1.73 m^2^ poses a substantial risk of progression to ESRD [37]. ACEI or ARB is effective for long-term renal survival of advanced IgAN patients of which most of their histological findings contain chronic glomerular abnormalities [38]. In future studies, we aim to clarify whether urinary C-megalin is a useful biomarker to determine the effectiveness of ACEI or ARB treatment. We also need to examine the levels of urinary C-megalin before and after the treatment in IgAN patients.

In conclusion, the levels of urinary C-megalin were correlated with mesangial hypercellularity and chronic extracapillary abnormalities in IgAN patients. There is a possibility that urinary C-megalin is an independent predictor of disease progression of IgA nephropathy. Possibly, urinary C-megalin is also a marker of glomerular abnormalities in MN. Further studies are needed to elucidate the molecular mechanisms of increased urinary excretion of C-megalin in patients with glomerular diseases.

## Supporting Information

S1 Table **Criteria for selection of normal control individuals among healthy volunteers.**
(PDF)

S2 Table **Stepwise multiple regression analysis of levels of eGFR with relevant factors.**
(PDF)

## References

[1] Saito A, Pietromonaco S, Loo AK, Farquhar MG (1994) Complete cloning and sequencing of rat gp330/“megalin,” a distinctive member of the low density lipoprotein receptor gene family. Proc Natl Acad Sci U S A 91:9725–9729. 7937880 10.1073/pnas.91.21.9725 PMC44889

[2] Hjälm G, Murray E, Crumley G, Harazim W, Lundgren S, et al (1996) Cloning and sequencing of human gp330, a Ca(2+)-binding receptor with potential intracellular signaling properties. Eur J Biochem 239:132–137. 8706697 10.1111/j.1432-1033.1996.0132u.x

[3] Saito A, Sato H, Iino N, Takeda T (2010) Molecular mechanisms of receptor-mediated endocytosis in the renal proximal tubular epithelium. J Biomed Biotechnol 2010:403272. 20011067 10.1155/2010/403272 PMC2789548

[4] Leheste JR, Rolinski B, Vorum H, Hilpert J, Nykjaer A (1999) Megalin knockout mice as an animal model of low molecular weight proteinuria. Am J Pathol 155:1361–1370. 10514418 10.1016/S0002-9440(10)65238-8 PMC1867027

[5] Saito A, Kazama JJ, Iino N, Cho K, Sato N, et al (2003) Bioengineered implantation of megalin-expressing cells: a potential intracorporeal therapeutic model for uremic toxin protein clearance in renal failure. J Am Soc Nephrol 14:2025–2032. 12874456 10.1097/01.asn.0000078804.98322.4a

[6] Birk HW, Piberhofer S, Schütterle G, Haase W, Kötting J, et al (1991) Analysis of Na+-D-glucose cotransporter and other renal brush border proteins in human urine. Kidney Int 40:823–837. 1762286 10.1038/ki.1991.282

[7] Bachinsky DR, Zheng G, Niles JL, McLaughlin M, Abbate M, et al (1993) Detection of two forms of GP330. Their role in Heymann nephritis. Am J Pathol 143:598–611. 8342605 PMC1887039

[8] Kounnas MZ, Chappell DA, Strickland DK, Argraves WS (1993) Glycoprotein 330, a member of the low density lipoprotein receptor family, binds lipoprotein lipase in vitro. J Biol Chem 268:14176–14181. 7686151

[9] Thrailkill KM, Nimmo T, Bunn RC, Cockrell GE, Moreau CS, et al (2009) Microalbuminuria in type 1 diabetes is associated with enhanced excretion of the endocytic multiligand receptors megalin and cubilin. Diabetes Care 32:1266–1268. 19366958 10.2337/dc09-0112 PMC2699744

[10] Ogasawara S, Hosojima M, Kaseda R, Kabasawa H, Yamamoto-Kabasawa K, et al (2012) Significance of urinary full-length and ectodomain forms of megalin in patients with type 2 diabetes. Diabetes Care 35:1112–1118. 22410816 10.2337/dc11-1684 PMC3329833

[11] Barratt J, Feehally J (2011) Primary IgA nephropathy: new insights into pathogenesis. Semin Nephrol 31:349–360. 21839368 10.1016/j.semnephrol.2011.06.006

[12] Berger J, Hinglais N (1968) Intercapillary deposits of IgA-IgG. J Urol Nephrol (Paris) 74:694–695. 4180586

[13] Haas M (1997) Histologic subclassification of IgA nephropathy: a clinicopathologic study of 244 cases. Am J Kidney Dis 29:829–842. 9186068 10.1016/s0272-6386(97)90456-x

[14] Lai KN, Leung JC, Chan LY, Saleem MA, Mathieson PW, et al (2009) Podocyte injury induced by mesangial-derived cytokines in IgA nephropathy. Nephrol Dial Transplant 24:62–72. 18685143 10.1093/ndt/gfn441

[15] Chan LY, Leung JC, Tsang AW, Tang SC, Lai KN (2005) Activation of tubular epithelial cells by mesangial-derived TNF-alpha: glomerulotubular communication in IgA nephropathy. Kidney Int 67:602–612. 15673307 10.1111/j.1523-1755.2005.67116.x

[16] Boyd JK, Cheung CK, Molyneux K, Feehally J, Barratt J (2012) An update on the pathogenesis and treatment of IgA nephropathy. Kidney Int 81:833–843. 22318424 10.1038/ki.2011.501

[17] Shigematsu H (1997) Histological grading and staging of IgA nephropathy. Pathol Int 47:194–202. 9103209 10.1111/j.1440-1827.1997.tb04480.x

[18] Roberts IS, Cook HT, Troyanov S, Alpers CE, Amore A, et al (2009) The Oxford classification of IgA nephropathy: pathology definitions, correlations, and reproducibility. Kidney Int 76:546–556. 19571790 10.1038/ki.2009.168

[19] Cattran DC, Coppo R, Cook HT, Feehally J, Roberts IS, et al (2009) The Oxford classification of IgA nephropathy: rationale, clinicopathological correlations, and classification. Kidney Int 76:534–545. 19571791 10.1038/ki.2009.243

[20] Asao R, Asanuma K, Kodama F, Akiba-Takagi M, Nagai-Hosoe Y, et al (2012) Relationships between levels of urinary podocalyxin, number of urinary podocytes, and histologic injury in adult patients with IgA nephropathy. Clin J Am Soc Nephrol 7:1385–1393. 22700887 10.2215/CJN.08110811 PMC3430952

[21] Matsuo S (2011) Clinical guides for immunoglobulin A (IgA) nephropathy in Japan, third version. Jpn J Nephrol 53:123–135. 21516693

[22] Matsuo S, Imai E, Horio M, Yasuda Y, Tomita K, et al (2009) Revised equations for estimated GFR from serum creatinine in Japan. Am J Kidney Dis 53:982–992. 19339088 10.1053/j.ajkd.2008.12.034

[23] Mundel P, Heid HW, Mundel TM, Krüger M, Reiser J, et al (1997) Synaptopodin: an actin-associated protein in telencephalic dendrites and renal podocytes. J Cell Biol 6:193–204. 10.1083/jcb.139.1.193 PMC2139823 9314539

[24] Tanuma A, Sato H, Takeda T, Hosojima M, Obayashi H, et al (2007) Functional characterization of a novel missense CLCN5 mutation causing alterations in proximal tubular endocytic machinery in Dent's disease. Nephron Physiol 107:87–97. 10.1159/000111253 18025833

[25] Kerjaschki D, Horvat R, Binder S, Susani M, Dekan G, et al (1987) Identification of a 400-kd protein in the brush borders of human kidney tubules that is similar to gp330, the nephritogenic antigen of rat Heymann nephritis. Am J Pathol. 129:183–91. 2444109 PMC1899684

[26] Nangaku M, Shankland SJ, Couser WG (2005) Cellular response to injury in membranous nephropathy. J Am Soc Nephrol. 16:1195–204. 15800119 10.1681/ASN.2004121098

[27] Lai KN, Tang SC, Leung JC (2011) Recent advances in IgA nephropathy–the glomerulopodocytic-tubular communication. Adv Otorhinolaryngol 72:40–44. 21865686 10.1159/000324593

[28] Lai KN, Leung JC, Chan LY, Saleem MA, Mathieson PW, et al (2008) Activation of podocytes by mesangial-derived TNF-alpha: glomerulo-podocytic communication in IgA nephropathy. Am J Physiol Renal Physiol 294:F945–955. 18256312 10.1152/ajprenal.00423.2007

[29] Tam KY, Leung JC, Chan LY, Lam MF, Tang SC, et al (2009) Macromolecular IgA1 taken from patients with familial IgA nephropathy or their asymptomatic relatives have higher reactivity to mesangial cells in vitro. Kidney Int 75:1330–1339. 19340088 10.1038/ki.2009.71

[30] Matsusaka T, Xin J, Niwa S, Kobayashi K, Akatsuka A, et al (2005) Genetic engineering of glomerular sclerosis in the mouse via control of onset and severity of podocyte-specific injury. J Am Soc Nephrol 16:1013–1023. 15758046 10.1681/ASN.2004080720

[31] Gonzalez-Villalobos R, Klassen RB, Allen PL, Navar LG, Hammond TG (2005) Megalin binds and internalizes angiotensin II. Am J Physiol Renal Physiol 288:F420–427. 15467006 10.1152/ajprenal.00243.2004

[32] Saito A, Takeda T, Hama H, Oyama Y, Hosaka K, et al (2005) Role of megalin, a proximal tubular endocytic receptor, in the pathogenesis of diabetic and metabolic syndrome-related nephropathies: protein metabolic overload hypothesis. Nephrology (Carlton) 10 SupplS26–31. 16174284 10.1111/j.1440-1797.2005.00453.x

[33] Lai KN, Chan LY, Leung JC (2005) Mechanisms of tubulointerstitial injury in IgA nephropathy. Kidney Int Suppl 94 S110–115. 10.1111/j.1523-1755.2005.09426.x 15752226

[34] Grgic I, Campanholle G, Bijol V, Wang C, Sabbisetti VS, et al (2012) Targeted proximal tubule injury triggers interstitial fibrosis and glomerulosclerosis. Kidney Int 82:172–183. 22437410 10.1038/ki.2012.20 PMC3480325

[35] Hasegawa K, Wakino S, Simic P, Sakamaki Y, Minakuchi H, et al (2013) Renal tubular Sirt1 attenuates diabetic albuminuria by epigenetically suppressing Claudin-1 overexpression in podocytes. Nat Med 19:1496–1504. 24141423 10.1038/nm.3363 PMC4041199

[36] Peters HP, van den Brand JA, Wetzels JF (2009) Urinary excretion of low-molecular-weight proteins as prognostic markers in IgA nephropathy. Neth J Med 67:54–61. 19299847

[37] Wyatt RJ, Julian BA (2013) IgA nephropathy. N Engl J Med 368:2402–2414. 23782179 10.1056/NEJMra1206793

[38] Moriyama T, Amamiya N, Ochi A, Tsuruta Y, Shimizu A, et al (2011) Long-term beneficial effects of angiotensin-converting enzyme inhibitor and angiotensin receptor blocker therapy for patients with advanced immunoglobulin A nephropathy and impaired renal function. Clin Exp Nephrol 15:700–707. 21625892 10.1007/s10157-011-0455-8

