# Supplementary material for: Significance of Urinary Full-Length Megalin in Patients with IgA Nephropathy
Source: PLoS One. 2014 Dec 12;9(12):e114400. doi: 10.1371/journal.pone.0114400 (PMC4264761; doi:10.1371/journal.pone.0114400)
Supplement: S1 Table — Criteria for selection of normal control individuals among healthy volunteers. (PDF) [file pone.0114400.s001.pdf]

Table S1 Criteria for selection of normal control individuals among healthy volunteers.

Medical parameters	cut-off values
BMI(kg/m^2)	≤ 24.0
Waist (cm)	(Men) ≤ 85, (Women) ≤ 90
Systolic blood pressure(mmHg)	< 130
Diastolic blood pressure(mmHg)	< 85
Total cholesterol (mg/dL)	< 220
Triglycerides(mg/dL)	< 150
LDL cholesterol(mg/dL)	< 140
HDL cholesterol(mg/dL)	≥ 40
AST(U/L)	≤ 40
ALT(U/L)	≤ 40
γ-GT(U/L)	(Men) ≤ 70, (Women) ≤ 30
Uric acid(hg/dL)	≤ 7.0
Serum creatinine(mg/dL)	(Men) < 1.05, (Women) < 0.8
eGFR(mL/min/1.73m^2)	60 ≤

AST, aspartate transaminase; ALT, alanine aminotransferase;
γ-GT, γ-glutamyl transpeptidase
