# Supplementary material for: Significance of Urinary Full-Length Megalin in Patients with IgA Nephropathy
Source: PLoS One. 2014 Dec 12;9(12):e114400. doi: 10.1371/journal.pone.0114400 (PMC4264761; doi:10.1371/journal.pone.0114400)
Supplement: S2 Table — Stepwise multiple regression analysis of levels of eGFR with relevant factors. (PDF) [file pone.0114400.s002.pdf]

Table S2 Stepwise multiple regression analysis of levels of eGFR with relevant factors.

variables	IgA nephropathy patients(N=73)	
	β	*p* value
C-megalin（pmole/g Cr）	-	-
β_2-MG（μg/g Cr）	-	-
α_1-MG（mg/g Cr）	-	-
NAG（IU/g Cr）	-0.27	0.004
